# B cells in head and neck squamous cell carcinoma: current opinion and novel therapy

**DOI:** 10.1186/s12935-024-03218-3

**Published:** 2024-01-20

**Authors:** Xinyue Guo, Licheng Xu, Luan Nie, Chenyu Zhang, Yaohui Liu, Rui Zhao, Jing Cao, Linli Tian, Ming Liu

**Affiliations:** 1 Department of Otorhinolaryngology, The Second Affiliated Hospital of Harbin Medical University, Harbin, China; 2 Department of Otorhinolaryngology, The First Affiliated Hospital of Harbin Medical University, Harbin, China

**Keywords:** HNSCC, TME, B cell, Therapy, Prognosis

## Abstract

Head and neck squamous cell carcinoma (HNSCC) is a common malignant tumour. Despite advancements in surgery, radiotherapy and chemotherapy, which have improved the prognosis of most patients, a subset of patients with poor prognoses still exist due to loss of surgical opportunities, postoperative recurrence, and metastasis, among other reasons. The tumour microenvironment (TME) is a complex organization composed of tumour, stromal, and endothelial cells. Communication and interaction between tumours and immune cells within the TME are increasingly being recognized as pivotal in inhibiting or promoting tumour development. Previous studies on T cells in the TME of HNSCC have yielded novel therapeutic possibilities. However, the function of B cells, another adaptive immune cell type, in the TME of HNSCC patients has yet to be determined. Recent studies have revealed various distinct subtypes of B cells and tertiary lymphoid structures (TLSs) in the TME of HNSCC patients, which are believed to impact the efficacy of immune checkpoint inhibitors (ICIs). Therefore, this paper focuses on B cells in the TME to explore potential directions for future immunotherapy for HNSCC.

## Introduction

Head and neck squamous cell carcinoma (HNSCC) arises from the oral cavity, oropharynx, hypopharynx and larynx [1]. According to several epidemiological statistics, HNSCC is the 6th most common cancer worldwide, accounting for approximately 900,000 new cases and 400,000 deaths annually [1, 2]. The incidence in France, Central Europe, and Eastern Europe is high [3]. About 90% of head and neck carcinoma cases are squamous cell carcinoma. HNSCC commonly correlates with heavy use of tobacco, excessive alcohol consumption, or both. In addition, many viral infections, such as human papillomavirus (HPV), primarily HPV-16 [4], and Epstein–Barr virus, have been confirmed to influence HNSCC development and progression. It is important to note that HPV-positive (HPV+) HNSCC generally has a better prognosis than HPV-negative (HPV-) HNSCC [1, 5].

HNSCC comprises a highly heterogeneous and aggressive group of tumours. Currently, the treatment for HNSCC relies mainly on surgical resection, followed by adjuvant radiation or platinum-based chemotherapy plus radiation (chemoradiation or chemoradiotherapy) depending on the disease stage [6]. The five-year survival rate of patients with advanced HNSCC remains less than 50% after initial therapy. Unfortunately, approximately 50% of patients develop recurrence or metastasis following conventional therapy. Patients with partial recurrence or metastasis have always lost the chance to receive salvage therapy. Eventually, palliative systemic therapy is needed [7]. Hence, exploring new methods, assessing the prognosis, understanding the underlying molecular mechanisms and improving the prognosis of HNSCC patients are crucial.

In recent years, research on immune cells has led to the development of new treatment methods for autoimmune diseases [8–10]. Moreover, immunotherapy has ushered in a new era for cancer treatment. Immunotherapy by chimeric antigen receptor-modified T (CAR-T) cells has shown exciting clinical effects on haematological tumours at the earliest stage. Studies have already found that CAR-T cells targeting EGFR or HER2 have anti-tumour efficacy in HNSCC. However, there are many difficulties in CAR-T-cell therapy for solid tumours. One important aspect of this disease is the hostile tumour microenvironment [11–13]. This dilemma is also found with immune checkpoint therapy (ICT) for programmed cell death-1 (PD-1)-expressing cells. The American Food and Drug Administration (FDA) has approved ICIs to treatment patients with HNSCC, which include pembrolizumab and nivolumab. ICIs have been used as the primary treatment for HNSCC patients whose disease progressed during or after platinum-based chemotherapy [14–16]. However, the response rate to PD-1/PD-1 ligand (PD-L1) inhibitors for recurrent or metastatic HNSCC has been disappointingly low, ranging from 13.3 to 17.9% in clinical trials [14]. Therefore, exploring potential antigen targets in HNSCC immunotherapy has always been an effective way to find better strategies [17]. Different antigens can be used to induce different levels of antitumour immune responses in the tumour microenvironment (TME). For example, melanoma-associated antigen and New York oesophageal squamous cell carcinoma-1, which are tumour-associated antigens [18], and the Epstein–Barr virus of viral antigen are associated with poor prognosis in HNSCC patients.

Regarding existing HNSCC immunotherapy, we can determine CAR-T-cell therapy and ICT are mainly targets T cells. Although there has been much progress in research on T cells, the greatest challenge is how to improve the efficacy of immunotherapy and benefit patients. B cells, also known as classical immune cells in the TME, are gradually being recognized as closely related to the response rate to PD-1 therapy [19, 20], even exceeding the scope explained by tumour-infiltrating T cells (TIL-T cells). An increasing number of studies suggest that B cells and tertiary lymphoid structures (TLSs) may have enormous antitumour potential [21]. Several scholars have noted the potential value of B-cell therapy in HNSCC [22]. For these reasons, we focused on the current status of B cells in the TME of HNSCC, exploring promising new options for HNSCC therapy and investigating the potential to increase the response rate to immunotherapy. By doing so, patients with HNSCC can benefit from the perspective of immunotherapy.

## B cells and HNSCC

B cells have been implicated in influencing the prognosis of patients with various tumours, either by promoting tumour progression or exerting tumour-suppressive effects [23]. Given that HPV infection is a known causative factor in HNSCC, researchers have observed intriguing characteristics of the humoral immune response and antigen specificity within the TME in HPV + HNSCC. Single-cell RNA sequencing analysis of B cells in the TME revealed that the tumour-infiltrating lymphocytes (TILs) in HPV + HNSCC patients includes germinal centre B cells (GCBs), activated B cells (ABCs) and antibody-secreting B-cell subsets. In addition, B cells and plasma cells (PCs) in the TME can improve patient prognosis by inhibiting antitumour immune responses through multiple mechanisms. Furthermore, increased HPV-specific antibody titres are associated with improved overall survival and a reduced risk of recurrence in patients with HPV + HNSCC [24, 25]. HPV serum antibodies have been described as predictors of survival and disease progression in patients with HPV + squamous cell carcinoma of the oropharynx [26]. Transcriptional data through multispectral immunofluorescence analyses, which evaluated cell‒cell communication, revealing significant differences in B-cell subsets between patients with HPV-HNSCC, HPV + HNSCC and healthy donors [27].

In contrast to HPV + TILs, in HPV-TILs, B cells are commonly found in the form of PCs or switched memory B cells. The predominance of PCs or early switched memory B cells in HPV- TILs may be attributed to the lack of CD4 + follicular helper T (TFH) cell support, which is potentially a consequence of the molecular differences between HPV- and HPV + tumours [5]. GCB is present across various stages of progression through germinal centre reactions in HPV + HNSCC but that B cells are present in fewer numbers and in nongerminal centre states in HPV- HNSCC. These observations highlight a spectrum of differences in immune lineages between HPV- and HPV + HNSCC. In several instances, CD4 + T cells are likely to participate in interactions with B cells. Researchers have found tertiary lymphoid structures (TLSs) in regions that contain high density of B cells. TLSs have been associated with improved survival across many cancer types [5]. These conclusions suggest that the phenotype and quantity of B cells in HPV + HNSCC patients may be one of the reasons for their better prognosis.

## B cells in the TME

The TME is defined as a complex and rich multicellular environment [28]. This environment typically includes various immune cells, such as adaptive immune cells, natural killer cells, mast cells, neutrophils, dendritic cells, tumour-associated macrophages, and myeloid-derived suppressor cells. Additionally, it includes stromal cells, the extracellular matrix, and various secreted molecules. Interactions between these cells and tumour components, along with complex regulatory networks, play a role in promoting or suppressing tumour progression, invasion, and metastasis [29]. Over the last decade, several studies have shown the essential role of adaptive immune cells, especially T cells, in antitumour immune responses [30, 31].

B lymphocytes, another important adaptive immune cell type found in the TME, are responsible for secreting antibodies and antigen presentation, thereby constituting the humoral immune response. However, our understanding of their function and role in the TME remains incomplete [32, 33]. There is significant heterogeneity in the function and surface immunophenotype of B-cell populations in the TME. This phenomenon is partly due to the lack of description of surface markers that define B-cell subsets [34]. Increasing evidence suggests that B cells may play a vital role in promoting or inhibiting tumour progression in the TME through processes such as antibody or cytokine production, costimulation and antigen presentation, direct cytotoxicity, interactions with myeloid cells. For example, Yong Wang et al. reported that myeloid-derived suppressor cells affect B-cell differentiation to promote tumour evasion of immune surveillance via transforming growth factor β-mediated IL-7 and downstream STAT-5 signalling pathways in lung cancer [35]. Such studies focusing on B cells open up the possibility of more effective antitumour immunotherapy [36].

## B cells in the TME in HNSCC (Yin and Yang)

**Fig. 1:**
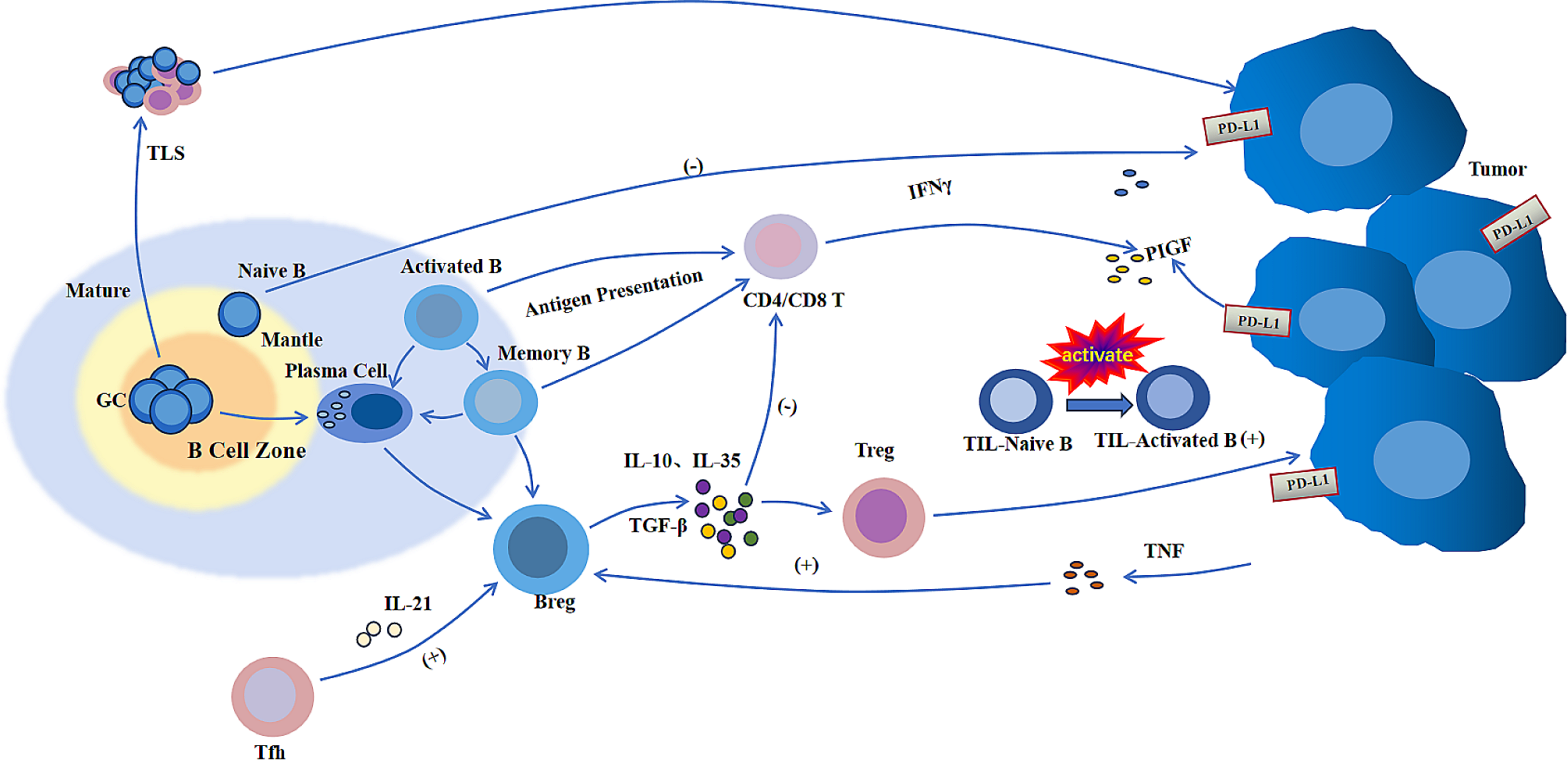
Different subtypes of B cells in the TME influence tumour growth through intercellular communication, cytokine secretion, etc. TLSs: tertiary lymphoid structures; GC: germinal centre; Tfh: follicular helper T cell; IL-21: interleukin-21; Bregs: regulatory B cell; TGF-β: transforming growth factor-β; IL-10: interleukin-10; IL-35: interleukin-35; Treg: regulatory T cell; INFγ: interferon γ; TIL-naive B: tumour infiltrating lymphocyte-naive B cell; PIGF: placental growth factor; PD-L1: programmed cell death-ligand 1; TNF: tumour necrosis factor

Based on the results of existing studies, it can be concluded that different subtypes of B cells play various roles in regulating the TME and can significantly impact patient prognosis. Animal models have shown that the growth of multiple murine tumour cell lines is decreased or even eliminated in animals lacking B cells (Jh-/-) [37]. Conversely, studies have reported an association between improved prognosis and an increased presence of B cells in patients with melanoma, sarcoma, breast cancer, oesophageal cancer [38]. Similar results have been observed in HNSCC, a positive correlation between the number of B cells and better prognosis [32]. Therefore, it may be inaccurate to judge the prognosis of a patient directly by the absolute number of B cells, possibly because different subtypes of B cells exert diametrically opposing effects on the tumour. To further elucidate the role of B cells in HNSCC, we elaborate on relevant research on B-cell types based on existing research. (Fig. 1)

### Naive B cells

As key cells for the formation of functional GCs, naive B cells have emerged as potential biomarkers for assessing response to PD-1 therapy. In melanoma patients, it has been observed that nonresponders to anti-PD1 and/or anti-CTLA4 agents have a greater abundance of naive B cells than responders. Naive B cells must be pulled into the GC to form functional GCs. Some studies indicate that high tumour-infiltrating B cells (TIL-B cells) and GCs are associated with favourable prognosis. A.T. Ruffin et al. proposed that driving naive TIL-B cells towards activated and germinal centre (GC) phenotypes may be a complementary strategy to enhance existing immunotherapeutic approaches [33].

### Memory B cells and activated B cells

In the early stages of the immune response, activated B cells undergo differentiation into memory cells and PCs through the germinal centre reaction [39]. Memory B cells, which are primarily responsible for the secondary immune response, can rapidly and effectively respond upon exposure to the corresponding antigen, amplifying the immune response [32, 33]. Therefore, it might contribute to antitumour immunity [40]. In both HPV + and HPV- HNSCC patients, there is an increase in the percentage of memory B cells in peripheral blood, accounted for 34% compared to 14%, respectively. Importantly, this increase is independent of tumour grade. Patients who respond to ICT exhibit an increased number of memory B cells [41, 42].

The heterogeneity of TIL-B cells is noteworthy in HNSCC, as elevated numbers of activated and memory B cells have been observed [41]. Additionally, A. Wieland et al. reported a subset of antigen-specific B cells known as ABCs (CD19 + CD20 + Ki-67+), they are found in peripheral blood after vaccination or infection. There are ABCs that are specific for tumour-associated viral antigens in the TME. Additionally, the presence of ABCs among HPV-specific antibodies suggests a prolonged and ongoing humoral immune response to these tumour-associated viral antigens. This may improve patient prognosis [26].

### Plasma cells (PCs)

PCs serve as the final effector cells in the B-cell differentiation pathway and can originate from activation of the marginal zone or follicular B cells, as well as from the germinal centre reaction involving memory B cells. Researchers have identified populations of PCs that exhibit immunosuppressive properties. For instance, CD19 + CD138 + IgA + cells play a critical role in tumour progression following treatment with the chemotherapeutic drug oxaliplatin [43]. Interestingly, low-dose oxaliplatin, which induces immunological cell death, was ineffective in the absence of B-cell depletion. These cells have also been found in therapy-resistant prostate cancer. However, the role and significance of PCs in the TME of HNSCC remain indeterminacy. Several studies have indicated a potential association between these cells and worse prognosis, but further research is needed to elucidate their precise functions and implications in HNSCC [33].

### Bregs and immunosuppression

Immunosuppressive mechanisms, including regulatory B cells (Bregs), have been identified in the TME. Numerous studies have demonstrated that B cells can exert suppressive effects on the adaptive immune response [44]. Bhan et al. specifically described a subset of B cells characterized by the CD1dhiCD21intIgMintCD23hi phenotype, which represents the first study on these inhibitory B-cell subsets [40, 45, 46]. Recent studies have associated the presence of Bregs with poor prognosis in cancer patients [43]. Bregs regulate immune tolerance by producing major immunosuppressive molecules, including interleukin-10, IL-35, and transforming growth factor β. These molecules inhibit expansion of T cells and other proinflammatory lymphocytes [47]. Such as, interleukin-10, a crucial element in the SHP-1-MAPK pathway, may affect tumour immunity through the regulation of molecules such as CD40 [48]. But there is still a lack of relevant research on HNSCC.

Studies involving humans and mouse models of HNSCC have identified subsets of B cells expressing CD25 with regulatory functions. Many Breg subsets with overlapping phenotypes and functions were also found [36]. In addition, different cells and molecules in the TME are involved in the regulation of the immunosuppressive ability of Bregs. Placental growth factor produced by tumour cells induces expression of TIL-B cells [49]. Interestingly, generation of TNF-α by tumour cells enhances expansion of Bregs [50]. IL-21, plays a critical role in the class switching of immunoglobulins by B cells and can increase the number of Bregs [51]. Interaction between PD-1 on B cells and PD-L1/2 expressed by other cells (mainly tumour cells and immune cells) can also induce expansion of Bregs. These findings highlight the necessity for further research on the interaction mechanisms between B cells and T-B cells in the TME.

### Tertiary lymphoid structures

The hallmark of mature TLSs is that they contain a large number of GCs surrounded by T cells and are filled with B cells [42].

Studies have shown a correlation between TLS density and tumour development or progression [52, 53]. In particular, TLSs with GCs in the TME correlate positively with the prognosis of patients [54]. The formation of GCs is needed for B-cell affinity maturation and antibody diversification [54]. Studies have shown that tumour-specific B may induce some molecular needed for TLS formation and maintenance [52]. The presence of B cells and TLSs can improve the prognosis of patients with HNSCC, especially in HPV + or treatment-naive patients [33]. TLSs are more strongly associated with improved prognosis in intratumoral environments than in peritumoral environments [55]. The presence of TLSs is related to an enhanced antitumour immune response [56]. TLSs may play a key role in cytotoxic chemotherapy, small-molecule inhibitor therapy and ICT [57]. Zhenghao Wu et al. reported that CD20 + CD22 + ADAM28 + BIR cells are present in TLSs of skin SCC and breast cancer patients can promote response to ICT [58]. Meantime, induction of TLS formation enhances the antitumour response to chemotherapy in pancreatic cancer [59].

Recent studies have preliminarily confirmed that the number of mature TLSs correlates positively with the prognosis of HNSCC patients. Thus, TLSs may be prognostic and predictive factors in cancer. This discovery opens up possibilities for the development of new immunotherapies that increase the antitumour immune response by targeting TLSs. For example, the RANK/LT pathway is involved in lymph node development, and targeting this pathway may regulate the development of TLSs by activating LTo cells to play an antitumour role. Lymphoid chemokines (such as CCL19–CCL21) may be good therapeutic targets for inducing TLS generation in melanoma and colorectal and lung cancer [53, 60].

## B-cell research on the TME in an animal model of HNSCC

### Current mouse model studies focus on the TME in HNSCC

Due to the challenges in obtaining samples and tumour heterogeneity in HNSCC, studies on B cells in the TME of HNSCC generally involve a few patients. Therefore, it is important to identify a suitable animal model for research. However, there is currently a lack of reliable mouse models for HNSCC research [61].

As in clinical research, investigations of immune cells in the TME of HNSCC have focused primarily on T cells. In 2019, YOU FU et al. utilized 4-nitroquinoline-1-oxide to induce squamous cell carcinoma in C57BL/6 mice. Their findings revealed many PD1 + T cells in the TME of JC12-xenografted tumours, suggesting that the JC1-2 tumour model may respond positively to PD-1/PD-L1 blockade [62].

In 2019, Sandra S. Jeske et al. injected the ADORA2A antagonist SCH-58,261 into a C3H/HeJ mouse model after inducing squamous cell carcinoma. This study demonstrated the presence of a novel adenosine-producing Breg population in the TME in both mice and humans. The ADO pathway in B cells may serve as a new therapeutic approach for HNSCC patients [63]. In another 2019 study, an AT-84-E7 mouse model was utilized to investigate the role of B-cell depletion in tumour growth. These findings showed that B-cell depletion actually promotes tumour growth [32]. Furthermore, in a mouse HNSCC model, Affara et al. demonstrated that B cells promote tumour development by depositing circulating immune complexes (produced by antibodies) in premalignant tissue [64]. In addition, several murine tumour models have shown that B cells can suppress T cells and promote tumour growth [63].

### Differences between animal models of HNSCC and human HNSCC

Studies in human solid tumours have shown that a high density of TIL-Bs is associated with favourable clinical prognosis, whereas studies in mouse models have shown that B cells have a tumour-promoting character [40].

These findings are based on experiments using µMt or Jh-/- mice, which are genetic models lacking B lymphocytes, or monoclonal antibodies to deplete B cells. The absence of B cells can enhance the role of other immune cells, such as natural killer cells and T cells. This finding suggested that B cells may have a negative regulatory effect on these immune cells, potentially promoting tumour development [37, 65].

### B cells enhance response to PD-1 therapy

As mentioned above, B cells have distinct subpopulations and perform multiple roles in the TME. In most large-scale population studies, the presence of B cells in cancer patients is associated with improved outcomes.

Furthermore, several research groups have highlighted the critical role of B cells in the response of cancer to ICIs [66]. B-cell-mediated T-cell activation and antibody generation play key roles in response to ICIs in a high-mutant-burden triple-negative breast cancer mouse model. These findings suggest that further investigations of B cells in the TME are potentially useful for enhancing the therapeutic efficacy of ICIs.

### Current state of treatment for TLSs and TIL-B

The potential role of TLSs and GC-B cells in tumour therapy is increasingly attracting attention. By conducting a more comprehensive characterization of TLSs, it may be identify a spectrum of TLS states with variations in cellular composition, location, maturation, and function [52]. When TLSs show GC activity (mature TLS), there is a significant increase in patient survival. Elucidating the molecular definitions of these different TLS states might enhance their value as prognostic and predictive markers in cancer patients. Notably, several scholars have reported that use of glucocorticoids may lead to a decrease in TLSs with germinal centres, which may have a negative impact on tumour patients [67].

When we comprehensively analysed current reports on TIL-B-cell therapy, we found that the effects of TIL-B-cell therapy on tumour growth in different studies were contradictory [68, 69]. TIL-B cells support antitumour immunity and promote immunotherapy responses by acting as APCs, producing antibodies and secreting cytokines. However, there is also evidence confirming the ability of TIL-B to promote tumour growth. Therefore, an urgent task is to accurately classify TIL-B cells through more standardized methods and explore in depth the mechanisms of action of B-cell subtypes (such as PCs) that may promote tumour growth. This type of research on TIL-B cells is likely to provide important assistance in assisting TIL-T-cell immunotherapy methods.

## Conclusion

Previous research on the TME of HNSCC has focused primarily on T cells and ICTs. In recent years, there have been advancements in understanding of B cells in the TME of HNSCC. Previous studies have highlighted the importance of different subtypes of B cells, which can both contribute to immune suppression and participate in tumour killing within the TME through secretion of cytokines and other mechanisms. However, many studies are limited by factors such as small sample sizes, variations in sampling locations, and differences in inclusion and exclusion criteria. These limitations can result in differing research conclusions. Furthermore, limitations of the data processing methods, such as the poor robustness and consistency of the tSNE or FltSNE dimensionality reduction algorithms, can contribute to varying conclusions regarding the function and role of B cells [70]. Therefore, additional studies with larger sample sizes, standardized protocols, and comprehensive criteria are needed to gain a deeper understanding of the role of B cells in the TME in HNSCC patients.

At present, research on B cells in the TME has led to proposals of many different subtype classification standards, but the generalizability of these subtypes has not been fully elucidated, and whether these subtypes can be further optimized has not been determined. Overall, different classification criteria present a challenge for researchers. Based on existing data, naive B cells and activated B cells may play a role in inhibiting tumour growth, while PCs, memory B cells and Bregs may promote tumour growth.

In the future, we anticipate that the study of B cells in the TME will benefit from the establishment of more comprehensive clinical and bioinformatics databases, allowing for analysis of larger sample sizes. This, in turn, may help in identifying interventions and target the internal immune suppression mechanisms within the TME. Investigating the intricate immune cell regulatory network led by B cells, which can inhibit tumour growth through intercellular communication, may offer new prospects for understanding and harnessing antitumour immunity. B cells, which have been preliminarily confirmed by revealing the specific mechanism of interaction between T cells and B cells, can be used to accurately screen potential beneficiaries of ICIs, enhance individualized plans for patients, further strengthen the clinical efficacy of ICT, and improve patient prognosis.

Additionally, the significant progress in the study of tumour-infiltrating TLSs within the TME should not be overlooked. Continued research on TLSs might lead to further benefits in HNSCC patients. By understanding and manipulating the role of TLSs in the TME, new therapeutic strategies can be developed to enhance antitumour immune responses and improve outcomes in patients with HNSCC.

## Data Availability

Because our research was a review study, we did not generate raw data.
